# Disability inclusion in Zambia’s government COVID-19 policies: a framework analysis

**DOI:** 10.1186/s12939-025-02656-3

**Published:** 2025-10-31

**Authors:** Queen E. Seketi, Nathaniel Scherer, J. Anitha Menon, Charles Michelo, Lena Morgon Banks, Virginia Bond

**Affiliations:** 1 Department of Epidemiology and Biostatistics, School of Public Health, The University of Zambia, Lusaka, Zambia; 2 International Centre for Evidence in Disability, London School of Hygiene and Tropical Medicine, London, UK; 3 Department of Psychology, School of Humanities and Social Sciences, The University of Zambia, Lusaka, Zambia; 4 School of Psychology, Curtin University Dubai, Dubai, United Arab Emirates; 5 Chreso University, Lusaka, Zambia; 6 Global Health Institute, Nkwazi Research University, Lusaka, Zambia; 7 Zambart, Lusaka, Zambia; 8 Department of Global Health and Development, Faculty of Public Health and Policy, London School of Hygiene & Tropical Medicine, London, UK

**Keywords:** COVID-19, Disability inclusion, Determinants of health, Equity, Framework analysis, Health policy, SDGs, Social justice, UNCRPD and Zambia

## Abstract

**Background:**

People with disabilities experienced disproportional health risks and systematic exclusion during the COVID-19 pandemic with particularly severe consequences such as poorer health outcomes and barriers to services in countries around the world. In Zambia, people with disabilities experienced income loss, stress, and additional barriers to accessing health services. This study aimed to analyse disability inclusion in national COVID-19 policies in Zambia.

**Methods:**

We conducted content policy analysis using framework analysis. Ten documents were analysed against eight equity-relevant dimensions of a typical disability inclusive, COVID-19 crisis response. We adapted the framework from Sakellariou and used Ritchie and Spencer’s five step thematic analysis. These national policies were published between March 2020 and December 2023. The documents were also scored against eight themes for policy provisions.

**Results:**

Disability inclusion was generally low. Although action was provided for disability inclusion in relation to accessible information, access to healthcare and education, financial support, and considerations for the needs of people facing multiple exclusion, information was often not detailed and did not cover all people with disabilities. Several themes on disability inclusion were neglected, including reasonable accommodation and disaggregated disability data.

**Conclusion:**

These findings underscore persistent structural barriers to equity for people with disabilities during public health crises. It highlights shortcomings by the Government of Zambia in promoting disability inclusion during the COVID-19 pandemic. There is need for improved provisions across all stages of policy design and implementation to strengthen equity and resilience in future public health emergencies.

## Introduction

The United Nations Convention on the Rights of Persons with Disabilities (UNCRPD) promotes disability inclusion and the rights of persons with disabilities globally [1]. Disability inclusion aims to consider the specific needs of people with disabilities to ensure that they have the same opportunities to participate in society on an equal basis. According to the World Health Organization (WHO), approximately 16% of the global population has a disability, representing 1.3 billion people, 80% of whom live in low- and middle-income countries [2]. “Persons with disabilities include those who have long-term physical, mental, intellectual or sensory impairments which in interaction with various barriers may hinder their full and effective participation in society on an equal basis with others” [1, p. 1]. People with disabilities are a marginalised group and are at greater risk of stigma and discrimination [3], poorer health outcomes [4, 5], poverty [6] and exclusion from community life because of environmental barriers [7]. Marginalisation and exclusion are due, in part, to the omission of the rights of people with disabilities in Government policy [8–11].

The protection and safety of people with disabilities in public health emergencies remains an important issue, as recognised in the 2015 Sendai Framework for Disaster Risk Reduction [12] and the United Nations Convention on the Rights of Persons with Disabilities (UNCRPD) [1]. Early in the COVID-19 pandemic, the World Health Organization (WHO) [13] and the International Disability Alliance [14] released guidelines for governments to address issues of disability inclusion. To reduce health disparities and unfavourable health outcomes and to promote and protect people with disabilities’ rights more broadly, during pandemic response and recovery efforts, governments were asked to take proactive steps to guarantee that the interests and concerns of people with disabilities, organisations of people with disabilities, and their families were considered. Despite this, during the COVID-19 pandemic, people with disabilities experienced higher mortality [5], poorer health outcomes [15, 16] and disproportionate social and economic impacts [17, 18].

Government policymaking and adequate resourcing of implementation are vital to promote disability inclusion and the rights of people with disabilities in pandemic response, including the right to accessible COVID-19 health information, accessible vaccination services, and continued rights to education. However, there is evidence that in many countries, COVID-19 policies and strategies lack disability inclusion. Across four South American countries (Argentina, Brazil, Chile, and Peru), a study revealed that, despite the wide range of COVID-19 policies implemented, many did not adequately consider the needs of people with disabilities and, in some cases, policies were actively detrimental to people with disabilities [19]. In South Africa, authors found limited considerations towards disability in pandemic decision-making and government action, due in part to resource constraints, limited knowledge of disability inclusion at the government level, and a lack of prioritisation [20].

In Zambia, approximately 7.7% of the population are people with disabilities [21]. Zambia has ratified the UNCRPD and has a disability legal and policy framework, including the Persons with Disabilities Act 2012 [22] and National Policy on Disability [23]. However, evidence shows limited implementation of disability inclusion in Zambia [24, 25]. Evidence has shown that people with disabilities in Zambia experienced health and social challenges during COVID-19. For example, a rapid health impact assessment found that families of children with disabilities had a major loss of income, housing instability, stress, child neglect and interrupted access to health services including rehabilitation (due to pandemic restrictions leading to closure of clinics at certain points) [26]. Further, qualitative research with people with disabilities and carers identified inaccessible COVID-19 information, exacerbated stigma and discrimination during the pandemic, and limited support at home and in healthcare settings [27].

At the beginning of the pandemic in March 2020, the government of Zambia declared COVID-19 a notifiable infectious disease (Public Health Act 1930, S.I.,2020/21) [28]. By April 2024, in Zambia, 349,304 COVID-19 cases and 4069 deaths had occurred [29]. The COVID-19 mortality data was not publicly disaggregated by disability. The government also issued several COVID-19 policies, guidelines and financial regulations. Some of these policies included the Education Contingency Plan for Novel Coronavirus [30] and the COVID-19 Workplace Safety and Health Guideline [31]. Policies are recognised as critical in shaping daily lives and influencing the structural drivers of health inequities, including those experienced by people with disabilities [2]. Yet, there is limited evidence on the extent to which government policies were disability inclusive across sectors in addressing inequities in access to socio-economic services during the COVID-19 pandemic. This issue of disability inclusion in policy is particularly important for low- and middle-income country contexts, where policy frameworks often intersect with longstanding systematic barriers. Examining disability inclusion in pandemic policy provides an opportunity not only to assess the adequacy of policy responses but also to generate lessons for building back better, such as strengthening health systems, and ensuring disability responsive policies in future crises. This study, therefore, used a framework analysis [32] to examine disability inclusion in the Government of Zambia’s COVID-19 policy response.

## Methods

### Study design

We conducted a policy content review of Zambian COVID-19 policies using framework analysis to examine disability inclusion.

### Policy document search and selection

We purposively searched for documents related to COVID-19 from the websites of all Zambian line ministries. We also directly contacted the Ministry of Health, Ministry of Education, and the Ministry of Community Development and Social Services in search of relevant policies. The inclusion criteria covered government policies, laws, plans, and statutory instruments relevant to the COVID-19 pandemic response in Zambia published between March 2020 and December 2023. Policies and strategies published by non-government agencies were excluded. Although speeches and media releases provide direction on government policy, we excluded these as not meeting inclusion criteria. We accessed all documents in their original language, namely English. To select policies, titles were screened against eligibility criteria by QES, MB, and VB. QES and NS subsequently screened policies against the full text for inclusion.

### Data analysis

We analysed policy content using five steps outlined by Ritchie and Spencer (1994) [32, 33] in their guidance on policy research, including *familiarisation; identifying a thematic framework; indexing; charting;* and *mapping and interpretation.*

Our method differed slightly, as we identified a thematic framework prior to analysis. We adapted the framework from Sakellariou et al. [19], who developed a thematic framework to examine disability inclusiveness in COVID-19 policies in South America. The framework was developed based on guidelines for disability inclusion during COVID-19 issued by international agencies, such as the International Labour Organization, the World Health Organisation [13], and the United Nations Office of the High Commissioner for Human Rights [34]. The framework outlines eight key areas for disability inclusion in the COVID-19 response (Table 1). The framework was modified to the Zambian context by the authors in four ways. Firstly, we removed “Protection of people living in residential settings” because it is unusual for many people with disabilities specifically to be in residential settings in Zambia and we targeted people with disabilities in the community as a deliberate decision to also align with the emphasis of moving towards Community -Based Inclusive Development (CBID). Secondly, a theme on “disability disaggregated data” was included to see if COVID-19 policy aligns with the 2015 Sendai Framework for Disaster Risk Reduction that calls for governments to provide mechanisms for disaggregation of data by sex, gender, and disability in emergency responses, to ensure that crisis response measures reach everyone, including people with disabilities [12]. Thirdly, the wording in the explanations of each theme in the framework was edited to fit the Zambian context. For example, in Zambia, both academic and vocational skills training are included under “Access to education.”

**Table 1:** Framework for policy content analysis, adapted from Sakellariou et al. 2020 [19]

Main Themes	Explanation
1. Accessible information	Provision of all information in accessible formats, including sign language translation, Braille script and easy read
2. Access to healthcare	Removal of financial barriers to care and measures to ensure equitable access to healthcare, including measures addressing disability-based discrimination
3. Access to education	Measures taken to ensure remote learning is fully accessible and to promote accessibility of both academic and vocational/informal schooling
4. Financial support	Provision of financial support (e.g. cash transfer, such as the Social Cash Transfer scheme) to people with disabilities and their family members if they had to stop working and measures taken to ensure financial support, including automatic extension of disability benefits
5. Reasonable accommodations for people with disabilities	Adjustments to public health measures to accommodate the needs of people with disabilities, including flexibility in restrictions on movement in public spaces
6. Consideration of the needs of people with disabilities who face multiple exclusions	Measures taken to protect people with disabilities who are at increased risk of social exclusion and poverty, such as women, children, homeless people and prisoners
7. Inclusion in decision-making process	Inclusion of people with disabilities and their representative organisations in advisory and decision-making bodies regarding pandemic governance
8. Disability disaggregated data	Enhancing the collection and interpretation of data disaggregated by income, gender, age and disability to generate disability disaggregated COVID-19 data to inform pandemic response.

Fourthly, we included a scoring system (Table 2). Policies were scored to examine the extent of disability inclusion against each theme. This scoring system was adapted from scoring systems from policy analyses of disability inclusion in health policies using EquiFrame [8, 10, 11, 35, 36]. A score of 3 or 4 indicates that the policy provided information on the theme that was actionable and operational. Under EquiFrame scoring, this is deemed “high quality.” Each theme was scored by the lead author (QES) and checked by the second author (NS). Discrepancies were discussed and resolved with support from co-authors. From the scoring, we calculated the number of policies receiving a score of at least 1 per theme and the average score per theme. The average score was calculated only from policies including a score of at least 1, as not all policies were expected to include information on each theme. As a final step, we extracted key disability inclusion and exclusion information relevant to each theme from all policies and mapped this information against the thematic framework.

**Table 2:** Qualitative scoring of disability inclusion

Evaluation aspect	Score
*Disability inclusion is not mentioned.*	Not Included
*Disability inclusion is mentioned only.*	1
*Disability inclusion is mentioned and explained*	2
*Actions identified to address disability inclusion.*	3
*Intention to monitor disability inclusion expressed*	4

### Ethical considerations

This study was approved by the University of Zambia Biomedical Research Ethics Committee (Ref: 3324–2022). No specific ethical issues were noted because all documents used are in the public domain and did not contain any personal information. Consent for publication is not applicable.

## Results

This section presents the findings according to the characteristics of the ten selected COVID-19 policy documents and results of the framework analysis according to the eight key themes of the adapted framework.

### Characteristics of the policies

i). Source and scope of implementation

We identified ten government policies and guidance published between 2020 and 2023 from five different sectors: Ministry of Health (*n* = 6), Ministry of Education (*n* = 1), Ministry of Community Development and Social Services (*n* = 1), Ministry of Labour and Social Security (*n* = 1), and Electoral Commission (*n* = 1). 6 out of 10 of the COVID-19 policy documents were from Ministry of Health. Regarding the scope of implementation, most of the COVID-19 policy documents (9 out of 10) were implemented nationwide. The exception was the guidance on the operation of Zambia’s Emergency COVID-19 Social Cash Transfer policy, implemented by the Ministry of Community Development and Social Services, which targeted 22 specific districts. Three of the policy documents were about COVID-19 vaccination uptake in Zambia.

ii). Disability terminology

Across the documents, we noted inconsistent language used in relation to disability. Some documents mentioned people with disabilities or children with disabilities, whereas others referenced the differently abled, those with special needs, or those with physical challenges. In some instances, this inconsistency regarding disability terminology was found within a single document. Various COVID-19 policy documents noted provisions for vulnerable populations, although it was poorly defined whether people with disabilities were included in this category.

iii). Frequency and range of scores across policies

In Table 3, we present the frequency of scores across all documents. The scoring of themes against each document is provided in Table 4. Each of the ten COVID-19 policy documents was scored once against each of the eight themes, and in total, there were 80 scores. If a document made no mention of a particular theme, we indicated ‘not included’, as per our adapted scoring. The vast majority of scores given to documents across all themes (66 out of 80) were scored as ‘not included’ (i.e. disability inclusion was not mentioned). Of the remaining scores with a value of 1 and above, 9 out of the 14 scores were scores of levels 3 (actions identified to address disability inclusion), while 5 out of 14 scores were at level 1 (disability inclusion mentioned only).

**Table 3:** Frequency of scores across policies

	Evaluation aspect	Score	Frequency of Score
	*Disability inclusion is not mentioned*	Not Included	66
	*Disability inclusion is mentioned only.*	1	5
	*Disability inclusion is mentioned and explained*	2	0
	*Actions identified to address disability inclusion.*	3	9
	*Intention to monitor disability inclusion expressed*	4	0
Total			80

**Table 4:** Scoring of COVID-19 policies by theme

Policy Name	Author (Year)	Accessible information	Access to healthcare	Access to education	Financial support	Reasonable accommodation	Considerations for people facing multiple exclusions	Inclusion in the decision-making process	Disability disaggregated data	Total number of themes per policy, Scoring 1+
The Public Health (Notifiable Infectious Diseases) (Declaration) Notice, Statutory Instrument 21	Ministry of Health (2020)	Not included	Not included	Not included	Not included	Not included	Not Included	Not included	Not included	0
Public Health (Infected Areas) (Coronavirus Disease 2019) Regulations, Statutory Instrument 22	Ministry of Health (2020)	Not included	Not included	Not included	Not included	Not included	Not included	Not included	Not included	0
Advocacy Communication and Social Mobilization (ACSM) Strategy to Promote Demand for COVID-19 Vaccines	Ministry of Health (2022)	(3)	Not included	Not included	Not included	Not included	Not Included	(3)	Not included	2
National COVID-19 Vaccine Deployment Strategy	Ministry of Health (2021)	(3)	(3)	Not included	Not included	Not included	Not included	Not included	Not included	2
Guidelines on involvement of Private Sector and Non-State Actors in the COVID-19 Vaccination Programme in Zambia	Ministry of Health (2021)	Not included	(3)	Not included	Not included	Not included	Not included	Not included	Not included	1
Education Contingency Plan for Novel Coronavirus	Ministry of General Education (2020)	(1)	Not included	(3)	Not included	(1)	(3)	Not included	Not included	4
Interim clinical guidance for management of patients with Coronavirus Disease 2019	Ministry of Health (2020)	Not included	(3)	Not included	Not included	Not included	Not included	Not included	Not included	1
Standard operating procedures against COVID-19	Electoral Commission (2021)	(1)	Not included	Not included	Not included	Not included	Not included	Not included	Not included	1
COVID-19 workplace safety and health guidelines	Ministry of Labour (2021)	Not included	Not included	Not included	Not included	Not included	Not included	Not included	Not included	0
Guidance on the operation for Zambia’s Social Cash Transfers during COVID-19	Ministry of Community Development and Social Services (2021)	(1)	Not included	Not included	(3)	Not included	(3)	Not included	Not included	3
Total number of COVID-19 policies including theme (Average score)		5 (2.5)	3 (3)	1 (3)	1 (3)	1 (1)	2 (3)	1 (3)	0 (0)	14

Source: Authors’ own scoring of ten COVID −19 policy documents

iv). Theme coverage

Per document, we defined theme coverage as the total number of themes scoring 1 and above. Theme coverage was highest in the Education Contingency Plan for Novel Coronavirus (COVID-19), published by the Ministry of General Education (2020). This document had covered four themes, including accessible information, access to education, reasonable accommodation and considerations for people who face multiple exclusions. However, only the access to education theme, out of four themes in the policy document, scored 3 (actions identified to address disability inclusion). The National COVID-19 Vaccine Deployment Strategy from the Ministry of Health, 2021, had two themes and both themes included in the policy document, namely accessible information and access to healthcare related to the Ministry of Health mandate, scored 3 (actions identified to address disability inclusion).

### Themes and level of disability inclusion

From the framework analysis, we analysed eight themes on disability inclusion in Zambian COVID-19 policies. Key themes were mentioned once at least in 7 out of 10 policy documents. As seen in Table 4, information on disability inclusion was available on accessible information, although this was not always actionable (average score 2.5, disability inclusion mentioned and explained). Overall, disability inclusion was better represented for five themes including access to healthcare, access to education, and financial support, considerations for people facing multiple exclusions, and inclusion in decision making process, within which actions to promote disability inclusion were stipulated across documents (average score 3, actions identified to address disability inclusion). Disability disaggregated information was not mentioned in any document. Information on disability inclusion was limited with regards to reasonable accommodation. For all themes, no specific actions on intention to monitor were recommended (score of 4). We present findings by theme. In Table 5, we have provided a summary of the outlined actions to support disability inclusion highlighted in the themes. Only information with a score of 3 has been included in the table.

**Table 5:** Actions to support disability inclusion (score of 3) by theme

Theme	Action to support disability inclusion
Theme 1: Accessible information	•Sign language interpretation for deaf and hard of hearing people. •Collaboration with OPDs and NGOs to promote accessible information
Theme 2: Access to healthcare	•Priority methods to deliver vaccines to people with disabilities, including fixed sites, mobile and outreach teams. •Financial contribution from private sector and non-state actors to support disability inclusion (cost of vaccines) •Clinical guidance for people with high-risk conditions, including HIV and diabetes
Theme 3: Access to education	•Developing relevant learning content •Identifying appropriate delivery platforms •Providing accessible teaching materials
Theme 4: Financial support	•Actions to ensure the safe and consistent delivery of the Social Cash Transfer
Theme 6: Considerations for people who face multiple exclusions	•Expansion of welfare benefits (Cash Transfers) •Priority for free personal protective equipment •Procurement and distribution of solar rechargeable radios to the poorest households
Theme 7: Inclusion in the decision-making process	•Worked with selected impairment representatives to oversee disability inclusion in the ministry of health (Recommends action to involve OPDs and NGOs targeting people with disability in governance mechanism)

Source: Authors’ own based on included COVID-19 policy documents

### Theme 1: Accessible information

Regarding *accessible informatio*n in COVID-19 policy documents, five out of ten included details on disability inclusion. Two out of the five policy documents provided specific actions on access to information for people with disabilities (score 3). The Advocacy Communication and Social Mobilisation (ACSM) Strategy to Promote Demand for COVID-19 Vaccines outlines the provision of sign language in videos and television presentations on COVID-19 vaccination. The document notes that the Zambia Deaf Society has been working with the Ministry of Health to promote accessibility. The document also recommends collaboration with non-governmental organisations (NGOs) and organisations of persons with disabilities (OPDs) to ensure the inclusion of other groups of people with disabilities, although there is no guidance on how to achieve this or what accessible measures may be needed. The National COVID-19 Vaccine Deployment Strategy specifies that information and education on COVID-19 vaccines must be provided in accessible formats, although specifics on what this means are not given. No document provides intention to monitor action points to make information on COVID-19, hygiene behaviours, and prevention measures accessible for people with disabilities.

### Theme 2: Access to healthcare

*Access to healthcare* is mentioned in three of the ten policy documents with an average score of 3 (actions identified to include disability inclusion). Firstly, the National COVID-19 Vaccine Deployment Strategy specifies that people with disabilities are at greatest risk of severe illness. The strategy outlines steps to ensure that people with disabilities receive the vaccine, including delivery of the vaccine by a combination of fixed site, mobile, and outreach teams to people confined at home or living in congregate settings. Second, the Guidelines on the involvement of Private Sector and Non-State Actors in the COVID-19 Vaccination Programme in Zambia states that private sector and non-state actors that contribute to vaccine procurement should contribute the cost of the vaccine per dose plus 10% to support access for the most vulnerable populations, including people with disabilities. Finally, the Interim Clinical Guidance for Management of Patients with COVID-19 does not make mention of people with disabilities broadly, but it does provide guidance for people with specific impairments and conditions associated with disability. Guidance includes information on home-based care and other special considerations. None of the policies makes mention of the removal of physical and financial barriers to healthcare or stigmatisation of people with disabilities in healthcare settings.

### Theme 3: Access to education

*Access to education* is represented in one policy document out of the ten COVID-19 policy documents, the Education Contingency Plan for Novel Coronavirus. The policy scored 3 in this theme, demonstrating at least some action toward disability inclusive education provision during the pandemic. The plan identifies children with disabilities as a priority group. Generally, the plan aims to ensure continuity of learning and notes that all activities should give due consideration to children with disabilities, although detail on what this means is not always provided. Activities outlined to promote learning for children with disabilities (the document defines these as “children with special educational needs”) include developing relevant content, identification of delivery platforms, and procurement and distribution of relevant teaching materials and equipment. There is no detail provided on what constitutes relevant content, delivery platforms, or teaching materials.

### Theme 4: Financial support

*Financial support* for people with disabilities is outlined in one out of the ten policy documents, the guidance on the operation for Zambia’s Social Cash Transfers during COVID-19. The policy document included some action towards disability inclusion and scored 3. Before COVID-19, the Social Cash Transfer (SCT) was provided to people with “severe” disabilities in Zambia who lived in a household meeting welfare criteria. If eligible, they receive double the standard cash transfer amount. The guidance for COVID-19 outlines methods to ensure that people with disabilities (and others eligible for the cash transfer) were supported to access the cash transfer safely and consistently during the pandemic. This includes methods to avoid COVID-19 infection in the case of physical cash payments.

### Theme 5: Reasonable accommodation

*Reasonable accommodation for people with disabilities* was largely missing across policies, with only one out of the ten policy documents covering the themes, with a score of 1 (disability inclusion is mentioned only). In this document, the Education Plan for Novel Coronavirus, disability inclusion was mentioned when presenting information on priority for face masks. The document noted that public health measures in education during COVID-19 should include people with disabilities (children with special needs). However, it did not identify actions related to reasonable accommodations such as flexibility in restrictions in movement in public places and the intention to monitor disability inclusion expressed.

### Theme 6: Consideration of the needs of people with disabilities who face multiple exclusions

Regarding *considerations of the needs of people with disabilities who face multiple exclusions* two of the ten policy documents identified actions to address disability inclusion (average score 3). Firstly, the Education Contingency for Novel Coronavirus, a Ministry of General Education (2020) policy, identified children with disabilities among people who face multiple exclusions and provides specific action regarding access to education:

*These efforts must aspire to reach all children in Zambia with an appropriate platform, with due consideration for girls, children with disabilities, refugees and migrants, and any other vulnerable groups.*

Secondly, the Guidance on the operation of Social Cash Transfers scored 3. It provides actions to promote continued access for people with disabilities who meet the criteria, being those from poorer social economic backgrounds (vulnerable groups) during the COVID-19 pandemic. The COVID-19 workplace safety and health guidelines from the Ministry of Labour (2021) provide for the evaluation of risk of exposure at workplaces for workers, including women with anomalies. However, as it was not clear if women with disabilities met criteria for “women with anomalies”, and it is not clear on actions for tacking multiple advantages, we have scored this document 0.

### Theme 7: Inclusion in decision-making process

*Inclusion in the decision-making process* was limited across the ten COVID-19 policy documents. Only one policy document, the Advocacy Communication and Social Mobilization (ACSM) Strategy to Promote Demand for COVID-19 Vaccines, contained information on including people with disabilities in COVID-19 decision-making with an average score of 3 (actions identified to address disability inclusion). It focussed on certain disability types, states:

*The Zambia Deaf Society has been championing these efforts and working together with Ministry of Health. There is also greater need for collaboration with other NGOs/CSOs who aim to reach and ensure inclusion of other groups (such as the blind or those with physical challenges) so that they can have up to date and accurate information and are also involved in community discussions.*

However, the policy gives no guidance on how to achieve and monitor disability inclusion in COVID-19 governance structures and/or emergency response teams.

### Theme 8: disability disaggregated data

*Disability disaggregated data* was not mentioned specifically across the ten COVID-19 policy documents. Although the ‘Education Contingency Plan for Novel Coronavirus’ indicated that monitoring of the action plan would be done, it did not mention any disaggregation by disability, only that data would be collected by social economic characteristics. The Standards and Curriculum directorate was assigned the role to monitor response and early recovery measures, and that monitoring tools would be developed. None of the policy documents mentioned alignment with existing information management systems, or with sustainable development goal indicators.

## Discussion

This paper set out to analyse the disability inclusiveness of Zambia’s Government COVID-19 policies using framework analysis. Across the 10 policy documents included, our analysis identified limited disability inclusion within policies and guidance on COVID-19, with 66 scores out of 80 scores showing “not included” and only 14 scores at 1 (disability inclusion mentioned) and above. Within themes, disability inclusion was often mentioned rather than explained with a few actions, especially with respect to access to information (average score of 2.5). Disability inclusion was mentioned, and some actions were provided on five themes, including access to healthcare, access to education, financial support, considerations for people facing multiple exclusions, and inclusion in the decision-making process (average score of 3, reflecting action identified to include disability inclusion). For these five themes, detail was often limited to ensure operationalisation. While some of the themes have actions, albeit with limited information, there were mentions only of disability inclusion without further policy actions provided for one theme, reasonable accommodation, (average score of 1). Finally, there was no mention of disability disaggregated data across the COVID-19 policy documents. These policy attributes have multiple and intersecting ways in which they continued to shape disability inclusion. In the discussion, we focus on three key issues from the policy analysis: policy omissions versus social justice, recognition of rights versus lack of corresponding action the need to move beyond general policy designs.

### Policy omissions versus social justice

First, regarding the issue of *policy omissions versus social justice*, our study findings on paucity of disability inclusive measures in government policy are consistent with several previous policy studies in Zambia conducted before the pandemic. Prior to the pandemic the issue of disability inclusion in Zambia was driven by implementation gaps between policy and practice [24, 37], often arising from a lack of understanding and coordination between those who plan and those who carry it out.

In Sub-Saharan Africa, the pattern of policy omissions is the same: For example, the African Union (AU) developed policies that recognise the rights of persons with disabilities. The expectation was that governments would in turn adopt and adapt these [38]. However, when these policies were adopted, they often lack implementation plans, budgetary allocations and enforcement mechanisms, which are needed to promote the implementation of disability inclusion [39]. This remained consistent across LMICs, where many ratified the UNCRPD and designed disability policies, yet the gap between policy and practice remained significant. In their analysis of the alignment of 14 national policies with the UNCRPD, Shikako et al. (2023) reported disparities across countries [40]. Similar to our study, Zandam et al. (2024) found better disability inclusion in COVID-19 vaccine drives in Zambia than on other domains [41].

Although Zambia has ratified the UNCRPD and put in place a Disability Act number 6 of 2012 [22], our analysis of COVID-19 policy in Zambia also reveals that scores of “not included” were actual omissions, signifying the absence of disability considerations in emergency settings such as pandemics. This suggests that policies were designed primarily for the general population. Measures specific to disability in COVID-19 policies were missing especially in the two Statutory Instruments [28, 42]; the Ministry of Labour policy [31] and the Electoral Commission policy [43]. Within themes related to main life areas during the pandemic, none mentioned intention to monitor disability inclusion. Disability Inclusive policies are important because they facilitate the removal of barriers to equal participation of people with disabilities in civil, political economic, social and cultural spheres while monitoring outcomes improves accountability [44–46].

To achieve systemic justice for people with disabilities during the pandemic, policies should have been more disability inclusive [13, 46–48], embracing social justice principles of access to resources, equity, participation and non-discrimination [49–51]. Studies have shown that without guidance, COVID-19 policy implementers at the grassroots level may have few tools and resources to work with, especially in low-resource settings [20]. Research shows that in addition to not having guidelines on disability inclusion, many health care workers, who are required to disseminate this health information, often lack knowledge on disability inclusion, are already overburdened with multiple tasks and, further, have been impacted directly and indirectly by the pandemic [52, 53].

This study underscores the need to reduce systemic injustice through disability inclusive policies, with limited disability inclusion in policies across sectors an ongoing issue in Zambia [24, 37, 54–56]. Recently, the United Nations Committee on the Rights of Persons with Disabilities made several recommendations to the Zambian government on how to improve disability inclusion [25]. They noted the need for improved disability inclusion in policies, the inclusion of people with disabilities in decision-making, and disability-disaggregated data with which to inform programming. The Committee also recognised the need for a better understanding of disability inclusion across all government entities and Ministries [25]. The government should prioritise adhering to the principles in the UNCPRD [57] and promoting the Sustainable Development Goals [58]. Regarding future pandemics, there are many participatory models and frameworks which government and stakeholders may use to co-design interventions that may address health and social disparities experienced by people with disabilities during a pandemic. For example, the PREparedness, REsponse and SySTemic transformation (PRE-RE-SyST) model includes actions to maintain essential services, provide accessible policy and public health information and engage with organisations of people with disabilities. Such models also recognise that governments need to address the structural and systemic causes of inequities experienced by people with disabilities that are exacerbated during a crisis such as COVID-19 [59], in keeping with international guidance [12, 13]. Another example is the Missing Billion Initiative’s System Level Assessment (SLA). The Initiative is working with global stakeholders and national governments to address inequities in health. Using a novel framework of disability-inclusive health systems, the Initiative supports governments to develop disability-inclusive health systems that provide access to people with disabilities on an equal basis to others. This includes action to provide training to health workers and ensure appropriate disability-focused health financing. Recently, the Initiative has partnered with the Ministry of Health in countries such as Chile, Nigeria and Mozambique. In Chile, the work resulted in formulation of a new National Policy on Inclusive Health for People with Disabilities. And in Nigeria, 40,000 health workers have been trained in disability inclusion across 36 states. This is an example of partnership and action that governments can take to strengthen disability inclusion and address inequities of access to services that are amplified during crises [60].

### Recognition of rights versus lack of corresponding action

A second key issue was the recognition *of rights without corresponding action*: Our policy analysis revealed that in some instances, the national COVID-19 policy at least mentioned disability. This is an indication of the Zambian government having recognised that people with disabilities needed considerations in the planning, response, and recovery plans, as directed by the WHO [13]. This finding agrees with a scoping review on COVID-19 policies in 18 countries in Africa, including Zambia. In their study, Kapiriri et al. found that people with disabilities were mentioned among the priority groups in COVID-19 policy in Zambia, but equity considerations were missing. This limitation was particularly evident in other LMICs [61]. The recognition of rights is a crucial step, but without corresponding action, these rights remain rhetorical. These shortcomings of the Zambian government to disability inclusion during COVID-19 through a lack of corresponding actions, like equitable resource allocations or delivery mechanisms, are consistent with other policy studies of four South American countries; studies in Australia; South Africa, and Chile [11, 19, 20, 62].

The lack of actionable measures in policies, in turn, impacts the ability of the system to be responsive to the actual needs of people with disabilities during the pandemic. For example, one of the issues identified was a lack of actionable policy measures on accessible communication for all impairment categories, to include people with cognitive, intellectual, developmental and/or communication impairments. This is contrary to article 9 of the UNCRPD on accessibility [51]. This means that people with intellectual impairments and autism were not catered for by government’s COVID-19 policy. The recognition of rights without major shifts in resources and mechanisms for delivery may partly explain lack of accessible information and negative effects experienced by many people with disabilities globally [46, 50, 63–65]. In Zambia, public health information was often disseminated and received in formats largely inaccessible to people with visual, hearing, and intellectual impairments [27]. While reasonable accommodation theme is implied in the labour policy [31], it lacked specificity and policies generally failed to incorporate disability inclusive safeguards. Another example is the Public Health (Infected Areas) (Coronavirus Disease 2019) Regulations under Statutory Instrument 22 prescribed penalties for non-compliance without recognising the barriers faced [42]. This is an example of an area where disability inclusion could have been provided for but clearly missed. This omission mirrors global critiques of pandemic responses that adopted coercion measures with potential negative consequences for people with disabilities [66]. According to the biopsychosocial model of disability embedded in the ICF, this lack of policy considerations is an environmental barrier to disability inclusion [67], contrary to the principle of non-discrimination [57]. In future, government and stakeholders should ensure that both targeted and mainstreamed actions [45] are co-designed with broader OPDs and family representatives of people with disabilities.

### The need to move beyond general designs

Lastly, the policy emphasis on general design over universal design further constrains access. Our findings are an indication of *the need for government policies to move beyond general designs* to embrace universal designs, by ensuring that public policies highlight non-discrimination and improve access for people with disabilities. One of the possible explanations for the policy visualisation having more scores for “not included” is that the COVID-19 policies in Zambia lacked the specificity and operational detail necessary for high level disability inclusion (level 4, intention to monitor disability inclusion expressed) [19]. Universal design has emerged as a gold standard for inclusion. This may include accessible physical infrastructure, accessible communication mechanisms, universal health coverage, inclusive eligibility criteria, in contrast to the general design [68–73]. These policies, if well implemented, could facilitate sustainable and disability inclusive access to and equal participation in most social economic spheres [74].

One notable area of policy inclusion in Zambia was the emergency social cash transfer programme, which expanded both coverage and support levels for vulnerable populations, including people with disabilities [75]. In financial support, and considerations for people facing multiple exclusions, cash benefits with top-ups (vertical expansion) and increased coverage of people with disabilities (horizontal expansion) were implemented [75]. However, coverage was uneven. People with disabilities who did not have a disability card, who did not reside in targeted areas, who were considered not poor, or who lost income due to pandemic related disruptions were often excluded [76]. This aligns with broader evidence indicating that persons with disabilities in LMICs were more susceptible to economic shocks during the pandemic but less likely to be protected by social insurance schemes. Neither were they always assessed for non-contributory social welfare support [77] and reported differential experiences [17, 18, 26, 27, 78–80]. The government should increase coverage of social protection interventions, as per Social Protection Floors Recommendation, 2012 [81–83], to cover many people with disabilities. This should be mainstreamed in national policy, with basic social security guarantees [83]. These improvements may echo Amartya Sen’s capability approach, which emphasises the expansion of real freedoms that individuals require to lead lives they value [84].

Zambia’s experience reflects a broader pattern in LMICs where universal design principles are implemented incrementally and inconsistently because of resource constraints [85], exacerbated by the COVID-19 pandemic [86]. The absence of universal designs and operational detail in crises responses undermines both first generation rights - participation, non-discrimination and second-generation rights - health, education with negative consequences like not considering special education supports when switching to remote schooling [77].

Another key contributing factor to this largely “exclusion within inclusion measures “in COVID-19 policy may have been the persistent poor availability of disability disaggregated data, which restricts evidence-based planning [17, 87]. Studies show that many policies in LMICs aggregate disability with other vulnerable groups such as women, children and older adults, and the general population, without recognising the unique challenges faced by people with disabilities. When disability indicators were included, the data were often outdated and insufficient to guide real time planning, for instance data from 2015 [21]. Moreover, the contributions of OPDs were often not acknowledged in many policy documents despite the UNCRPDs call for their participation. Many policies in LMICS are designed without the active participation of people with disabilities, which might lead to a disconnect between policy intentions, and the actual needs of the disability community. Sufficient engagement and involvement of expert OPDs during the policy phases have the potential to improve disability inclusive frameworks and in line with the mantra of “nothing about us without us” [51, 58, 87].

Zambia, like many LMICs, missed a policy window that could have enabled more transformative inclusion. According to Kingdon’s multiple streams theory, effective policy changes occurs when problem, policy and political streams converge [88]. Between 2020 and 2022, there was a policy window, during which policy makers were receptive to new ideas and policy changes during the pandemic. The problem stream involved growing awareness of the disproportionate impact of the COVID-19 on people with disabilities [13, 18, 26, 47, 79]. The policy stream (policy action) encompassed the development of disability-inclusive solutions, such as accessible vaccination packages and inclusive social protection programmes. Some actions were provided, example in access to health, access to education, and social protection. However, details were missing to ensure effective operationalisation, and this finding is in keeping with other policy analyses and scoping reviews [89]. Content was missing on policy initiatives and strategies to maintain disability inclusive access to healthcare, community and support networks, and training and education of healthcare professionals to better understand the needs of people with disabilities during the pandemic. On access to education, digital solutions to either initiate or maintain access to remote learning for people with disabilities were not covered, nor was intention to monitor actions on actual remote learning for people with disabilities. Before the pandemic, interventions on disability inclusive learning were already problematic; pandemic restrictions further pushed some learners with disabilities out of the school system through inadequate access to resources to participate and the inability to return to school because of falling behind in schoolwork.

In the future, multisectoral action is key because studies have shown that during pandemics, no one key actor is expected to shoulder all the policy burdens [2, 12, 13]. For example, the Disaster Management and Mitigation Unit, the Zambia National Public Health Institute, and the Zambia Agency for persons with disabilities must work together when planning response and recovery measures, so that interventions are synchronised to achieve equitable outcomes of pandemic interventions.

### Strengths and limitations

This is the first analysis of multisector government COVID-19 policies in Zambia using a disability inclusion lens. The study utilises a structured framework based on international guidance and used in other settings.

Regarding limitations, firstly, we did not measure the frequency of actions in a document. Whether a policy document provided 100 detailed actions or one action with limited information, it was scored 3. As noted in the results, policies often provided limited information to operationalise actions when scoring 3, and we encourage cautious interpretation on the strength of these policies towards actionable disability inclusion. Secondly, in real contexts, policy contents may overlap, especially when policymakers reference an earlier document as key to the interpretation and implementation of the new policy. For example, Statutory Instruments referenced consistency with previous provisions of the Public Health Act (1930), however, the Public Health Act was not analysed as COVID-19 policy in this paper because it was formulated in 1930. Thus, disability-inclusive provisions in this Act and as noted to be applicable to certain COVID-19 policies, were not assessed, which may underestimate the level of disability inclusion intended and interpreted by policy actors in Zambia. We did not analyse the policies of NGOs and faith-based organisations, as they operate hospitals, boarding schools and orphanages where people with disabilities may have been receiving services. Comparing these contexts could have shed light on how Government COVID-19 policies were translated into practice.

## Conclusion

While the role of policy in shaping disability inclusion is now widely recognised, Zambia’s COVID-19 response policies fell short of delivering a fully disability inclusive approach to response and recovery. Despite the fundamental rights being enshrined in international and national law, COVID-19 policies rarely incorporated substantial measures to eliminate barriers to participation in key aspects of life during the public health crisis. This gap reflects the persistence of negative societal perceptions of disability and has likely reinforced structural barriers leaving many people with disabilities vulnerable to continuing socioeconomic and health disparities, as well as new and emerging challenges. There is an imperative need for policy makers, public health practitioners, OPDs and other relevant stakeholders to take joint responsibility for developing and implementing long-term strategies that safeguard and advance disability rights within inclusive pandemic preparedness, response and recovery efforts. Such strategies should prioritise health equity, strengthen system resilience and ensure that people with disabilities are meaningfully involved at all stages of policy design and implementation in their respective fields. Future research should investigate the perspectives of disability service providers, identifying practical obstacles to the enacting disability-inclusive measures. In doing so, it is important that stakeholders co-create public health policies in partnership with specialized organizations representing people with disabilities to ensure that interventions are both contextually relevant, genuinely inclusive, and more sustainable.

## Data Availability

All relevant data abstracted or analysed during this study are included in the text and also available in the public domain.

## Declarations

### Disclaimer

All the views expressed in this article are those of the authors and are the product of research. The views expressed in this article do not reflect the official policy or position of any affiliated institution or those of the publisher. The authors are solely responsible for this article’s contents.

## Abbreviations

ACSM: Advocacy Community and Social Mobilisation
AU: African Union
CBID: Community Based Inclusive Development
CSO: Civil Society Organisation
COVID-19: Coronavirus Disease 2019
HIV: Human Immuno-Deficiency Virus
ILO: International Labour Organisation
LMICs: Lower Middle-Income Countries
NGO: Non-Governmental Organisation
OPDs: Organisations of People with Disabilities
PENDA: Programme for Evidence to inform Disability Action
SCT: Social Cash Transfer
SDGs: Sustainable Development Goals
UNCRPD: United Nations Convention on the Rights of Persons with Disabilities
WHO: World Health Organization
